#  Eye movements in developing readers: From basic research to classroom application. Parts of symposium 7 at the 20th European Conference on Eye Movements in Alicante, September 21, 2019 

**DOI:** 10.16910/jemr.12.7.10

**Published:** 2019-11-25

**Authors:** Alexandra Spichtig, Christian Vorstius, Ronan Reilly, Jochen Laubrock

**Affiliations:** Reading Plus, USA; Bergische Universität Wuppertal, Department of Psychology; Maynooth University, Ireland; Universität Potsdam, Germany

**Keywords:** reading, reading development, perceptual span development

## Abstract

Eye-movement recording has made it possible to achieve a detailed understanding of oculomotor and cognitive behavior during reading and of changes in this behavior across the stages of reading development. Given that many students struggle to attain even basic reading skills, a logical extension of eye-movement research involves its applications in both the diagnostic and instructional areas of reading education. The focus of this symposium is on eye-movement research with potential implications for reading education.

Christian Vorstius will review results from a large-scale longitudinal study that examined the development of spatial parameters in fixation patterns within three cohorts, ranging from elementary to early middle school, discussing an early development window and its potential influences on reading ability and orthography. Ronan Reilly and Xi Fan will present longitudinal data related to developmental changes in reading-related eye movements in Chinese. Their findings are indicative of increasing sensitivity to lexical predictability and sentence coherence. The authors suggest that delays in the emergence of these reading behaviors may signal early an increased risk of reading difficulty.

Jochen Laubrock’s presentation will focus on perceptual span development and explore dimensions of this phenomenon with potential educational implications, such as the modulation of perceptual span in relation to cognitive load, as well as preview effects during oral and silent reading --and while reading comic books.

**Video stream:** https://vimeo.com/362645755

